# Editorial: The Process Evaluation of Clinical Trials

**DOI:** 10.3389/fmed.2022.950637

**Published:** 2022-07-11

**Authors:** Hueiming Liu, Gerhard Andersson, Vinaya Manchaiah

**Affiliations:** ^1^Center Health Systems Science, The George Institute for Global Health, The University of New South Wales, Newtown, NSW, Australia; ^2^School of Public Health, University of Sydney, Sydney, NSW, Australia; ^3^Department of Behavioral Sciences and Learning, Department of Biomedical and Clinical Sciences, Linköping University, Linköping, Sweden; ^4^Division of Psychiatry, Department of Clinical Neuroscience, Karolinska Institute, Stockholm, Sweden; ^5^Department of Otolaryngology–Head and Neck Surgery, University of Colorado School of Medicine, Aurora, CO, United States; ^6^UCHealth Hearing and Balance, University of Colorado Hospital, Aurora, CO, United States; ^7^Virtual Hearing Lab, Collaborative Initiative Between University of Colorado School of Medicine and University of Pretoria, Aurora, CO, United States; ^8^Department of Speech-Language Pathology and Audiology, University of Pretoria, Pretoria, South Africa; ^9^Department of Speech and Hearing, Manipal College of Health Professions, Manipal Academy of Higher Education, Manipal, India

**Keywords:** clinical trials, process evaluation, qualitative, mixed-methods, randomized controlled trial, implementation science, implementation research

## Background: History of Process Evaluations

The history of clinical trials goes back a long way to 500 BC, though some credits French surgeon Ambrose pare for the first documented clinical trial involving treatment of wounds during sixteenth century (1). Since then, clinical trials have evolved tremendously and have now become the foundation of modern medical and healthcare practice, focusing on clinical outcomes. However, over the past decades there has been increasing interest in performing “process evaluations” of clinical trials of complex interventions (2). While the outcome evaluation focuses on whether a new intervention works, a process evaluation supplements our knowledge by providing an understanding of the causal mechanisms of the intervention, contextual factors, and implementation factors impacting on the outcomes (3).

Process evaluation methodology has evolved through the years (2). Previously, they were used to assess implementation through the analysis of quantitative process indicators. Subsequently, there was increasing recognition and the need for qualitative research alongside trials to provide a deeper understanding of the disease condition, acceptability of an intervention and implementation issues (4). Process evaluations were deemed particularly relevant during a negative trial result, as to whether there was either implementation or intervention failure, or both. However, there is also a growing recognition that using qualitative and quantitative data, and theoretical frameworks within process evaluations will help facilitate evidence to practice (5–7). Process evaluations can help address stakeholders' question of “Is this intervention acceptable, effective, affordable and feasible (for me or) for this population?” (7).

Key domains are summarized in UK Medical Research Council (MRC) process evaluation guidance (context, quality of implementation and mechanisms of the intervention), and also include concepts from established evaluation frameworks that have been used widely including: Reach, Effectiveness, Adoption, Implementation and Maintenance framework (RE-AIM) (8) and Linnan and Steckler (9). Although each is unique, there is some overlap, in their emphasis to enable research translation. The key concepts include: (i) reach and recruitment (i.e., investigating the extent to which the intervention as received by the targeted group), (ii) adoption (i.e., related to the delivery of the intervention, (iii) acceptability (i.e., extent to which participants find the intervention acceptable), (iv) implementation fidelity (i.e., extent to which intervention is delivered as planned), (v) maintenance (i.e., extent to which the intervention can be sustained over time after the clinical trial is over).

## Special Issue: Process Evaluations of Clinical Trials

This special issue builds on the emerging value and methodology of process evaluations. It includes nine manuscripts focusing on a range of interventions. Therefore, highlighting the transferability and value of process evaluations across types of interventions, and also in unpacking context from lower-middle income countries to high income countries with established health systems. Chu et al. presented the mixed-methods process evaluation of community-based dietary sodium reduction in Rural China. In another study, expectations regarding pragmatic trial design of integrative medicine for diabetes and kidney diseases among patients and physicians was evaluated and reported (Chan et al.). Four studies focused on process evaluation of telehealth interventions. Meijerink et al. presented process evaluation of online support program for hearing aid users. Beukes et al. and Biliunaite et al. provided process evaluation results of internet-based cognitive behavioral therapy for tinnitus and informal caregivers, respectively. Indraratna et al. presented the process evaluation of TeleClinical care for acute coronary syndrome and heart failure. Two studies also included implementation science approach. Riddell et al. evaluated the implementation and scalability of the Accredited Social Health Activists (ASHAs) led community-based support groups for hypertension in Rural India. In another study, Ouyang et al. provided the process evaluation of implementation trial on intracerebral hemorrhage. Finally, Wu et al. presented the comprehensive process evaluation of the pediatric drug clinical trials through a literature review.

The process evaluations in this collection are also conducted across different phases of the research cycle, from study design (Chu et al.), pilot/feasibility phase (Biliunaite et al.; Indraratna et al.), evaluation of the clinical trial (Chu et al.; Meijerink et al.; Beukes et al.; Riddell et al.; Ouyang et al.) including long term sustainability (Riddell et al.; Wu et al.). Therefore, highlighting the value of process evaluation findings to inform intervention design and optimize implementation. Moreover, while the use of theoretical frameworks is helpful in eliciting contextual determinants across individual, organizational and system, and policy levels, often the breadth and scope of them in literature can be daunting (3). Careful consideration of what theories are relevant would be helpful (10). For instance, in this special issue, for interventions that are related to individual behavioral change, health belief model used by Chu et al. or others such as behavior change wheel, or cognitive theories may be helpful. Normalization process theory that has a strong focus on understanding organizational behavior, was also used by Ouyang et al. the implementation study in stroke units for intracerebral hemorrhage.

## Where to From Here?

Indeed, as we reflect on the emerging value and methodology of process evaluations, it is worth noting its contributions to implementation science, as researchers endeavor to meet end-users' needs, understand what happened on the ground, and how to overcome implementation barriers. As we continue to invest in clinical trials to inform evidence-based medicine and policy, we recommend that we embed process evaluations throughout the research cycle, to examine for whom, how and why the clinical trial had its outcomes. This will require building capacity in mixed-methods, implementation science, stakeholder engagement/co-design of implementation strategies, which will require allocation of sufficient resourcing, budgeting, time and most importantly training those who are involved in performing clinical trials on process evaluation and implementation science elements. And in doing so, regardless of a positive or negative trial result, we will learn to improve our research and intervention design to meet local context and enable long term sustainability and scale up of effective interventions.

## Author Contributions

HL drafted the initial manuscript, with significant input from VM. All authors have reviewed the manuscript versions and approved its submission.

## Funding

HL is supported by a Program Grant Fellowship from The George Institute for Global Health.

## Conflict of Interest

The authors declare that the research was conducted in the absence of any commercial or financial relationships that could be construed as a potential conflict of interest.

## Publisher's Note

All claims expressed in this article are solely those of the authors and do not necessarily represent those of their affiliated organizations, or those of the publisher, the editors and the reviewers. Any product that may be evaluated in this article, or claim that may be made by its manufacturer, is not guaranteed or endorsed by the publisher.

## References

[1] Bhatt A. Evolution of clinical research: a history before and beyond James Lind. Perspect Clin Res. (2010) 1:6–10. 21829774 PMC3149409

[2] Moore GF Audrey S Barker M Bond L Bonell C Hardeman W. Process evaluation of complex interventions: Medical Research Council guidance. BMJ. (2015) 350:h1258. 10.1136/bmj.h1258 25791983 PMC4366184

[3] Liu H Mohammed A Shanthosh J News M Laba TL Hackett ML. Process evaluations of primary care interventions addressing chronic disease: a systematic review. BMJ Open. (2019) 9:e025127. 10.1136/bmjopen-2018-025127 31391188 PMC6687007

[4] Lewin S Glenton C Oxman AD. Use of qualitative methods alongside randomised controlled trials of complex healthcare interventions: methodological study. BMJ. (2009) 339:b3496. 10.1136/bmj.b3496 19744976 PMC2741564

[5] Curry LA Nembhard IM Bradley EH. Qualitative and mixed methods provide unique contributions to outcomes research. Circulation. (2009) 119:1442–52. 10.1161/CIRCULATIONAHA.107.742775 19289649

[6] McIlvennan CK Morris MA Guetterman TC Matlock DD Curry L. Qualitative methodology in cardiovascular outcomes research: a contemporary look. Circ Cardiovasc Qual Outcomes. (2019) 12:e005828. 10.1161/CIRCOUTCOMES.119.005828 31510771

[7] Skivington K Matthews L Simpson SA Craig P Baird J Blazeby JM. A new framework for developing and evaluating complex interventions: update of Medical Research Council guidance. BMJ. (2021) 374:n2061. 10.1136/bmj.n2061 34593508 PMC8482308

[8] Glasgow RE Vogt TM Boles SM. Evaluating the public health impact of health promotion interventions: the RE-AIM framework. Am J Public Health. (1999) 89:1322–7. 10.2105/AJPH.89.9.1322 10474547 PMC1508772

[9] Linnan L Steckler A. Process Evaluation for Public Health Interventions and Research. San Francisco, CA: Jossey-Bass (2002).

[10] Nilsen P. Making sense of implementation theories, models and frameworks. Implement Sci. (2015) 10:53. 10.1186/s13012-015-0242-0 25895742 PMC4406164

